# New metrics for governance in the era of earth observation data: Monitoring violations after wildfires

**DOI:** 10.1093/pnasnexus/pgae466

**Published:** 2024-10-16

**Authors:** Germana Corrado, Luisa Corrado, Fabio Del Frate, Davide De Santis, Francesca Marazzi

**Affiliations:** Department of Management and Law, University of Rome “Tor Vergata”, Rome 00133, Italy; Department of Economics and Finance, University of Rome “Tor Vergata”, Rome 00133, Italy; Department of Civil Engineering and Computer Science Engineering, University of Rome “Tor Vergata”, Rome 00133, Italy; Department of Civil Engineering and Computer Science Engineering, University of Rome “Tor Vergata”, Rome 00133, Italy; Department of Economics and Finance, University of Rome “Tor Vergata”, Rome 00133, Italy

**Keywords:** satellite earth observation, wildfires, illegal building detection, misgovernment

## Abstract

This study uses Earth observation data to measure illegal activities and investigates possible relationships with local governments. We have collected satellite images, digital maps, and geospatial data for over a decade to detect potential illegal constructions in protected burned forest areas in Sardinia, Italy. We create a database of buildings erected in these protected areas and connect it to administrative data on local election results. First, we examine the climatic and geographical factors that contribute to wildfires. Our findings indicate that fires tend to spread more in places with higher altitudes and greater distances from urban centers. This highlights the challenges that local authorities face in monitoring and intervening in areas that are less accessible. Next, we analyze the relationship between mayoral turnover and constructions erected in protected burned forests to highlight how this phenomenon influences voters’ preferences. We observe that citizens express their disapproval of these illegal activities through the ballot box. Violations in the years leading up to local elections increase the likelihood of a change in mayoral leadership within a municipality.

**Significance Statement**

The abundance of geospatial information provided by space agencies and local governments represents an unparalleled resource for evaluating hard-to-measure economic outcomes. Specifically, we use Earth observation data to identify potentially illegal constructions built in protected areas hit by wildfires. This approach allows us to create comprehensive datasets at a municipal level, providing valuable information on fire incidents and potential violations that may follow. Cross-referencing these data with administrative records of election results reveals a correlation between illegal construction activities and the turnover of local authorities. By identifying these patterns, governments can address the underlying causes of these illegal activities proactively. This enables them to ensure that their elected officials prioritize environmental protection through increased surveillance and law enforcement.

## Introduction

In recent years, the frequency and extent of wildfires have increased, posing significant challenges to local communities and governments. Adding to this complex issue is the presence of illegal construction activities seeking quick profits or settlement opportunities, which are difficult for local authorities to monitor, particularly in more remote and less accessible areas. In this paper, we use Earth Observation (EO) data to identify constructions that are likely to be unauthorized within fire-affected forest areas. Illegal construction in protected areas is a serious issue in developing countries due to the rapid population growth. However, it also represents an unresolved problem in some developed countries, such as Italy (1). Unlawful construction is a criminal offense in Italy, yet norms, penalties, and fines have not deterred this phenomenon, which is quite widespread across regions. The negative consequences of these illegal activities are both environmental and economic. On the one hand, infrastructures and buildings constructed in protected areas, such as natural reserves and forests, are significant causes of landscape degradation, which has even stronger consequences when perpetrated in areas predominately supported by tourism. On the other hand, illegal buildings are not listed in the land registry and, as such, their owners evade property taxes, thus reducing the revenue of local governments.

In places where illegal construction is widespread, remote sensing is a valuable technology for accurately identifying suspicious buildings (2). Earth observation data, including satellite images and aerial imagery, can be stored on globally shared portals managed by various space agencies, such as the National Aeronautics and Space Administration (NASA) and the European Space Agency (ESA). These images allow users to track changes in the environment as well as in human settlements. EO data are available with spatial coverage of the entire globe and, increasingly since the beginning of the century, with multiple images over the same geographical area, which can be acquired over time. This lays the ground for a wide range of applications and, more importantly, encourages cross-fertilization between EO technology and other disciplines. Furthermore, the recent Copernicus program (https://www.copernicus.eu/en) has enhanced the dissemination of EO data and encouraged inter-disciplinary collaboration based on the exploitation of satellite data.

In this regard, the amount of EO and geospatial data provided by space agencies and local authorities over the past decades represents an unprecedented source of information. The social sciences have used remote sensing in various fields of research. Applications include analyzing urbanization and the associated change in land cover (3, 4), estimating housing prices based on urban development and land availability (5, 6), and assessing socioeconomic differences associated with housing quality retrieved by satellite imagery (7–9). Other studies that are closer to ours use satellite data to study regional political processes (10). Furthermore, as Donaldson and Storeygard (11) have emphasized, the use of remote sensing data to identify individual buildings is an ongoing area of research. With advances in spatial and temporal resolution, an increased number of satellites, and enhanced algorithms, future studies hold the potential to globally monitor illegal land use, even at the level of individual plots.

Illegal buildings are often associated with a lack of efficiency, inadequate surveillance, and weak law enforcement by the local authorities, which can in turn have a significant influence on the preferences of voters. Casaburi and Troiano (12) examined the electoral response to the Ghost Buildings program, a national antitax evasion policy in Italy that used surveillance technologies to target buildings hidden from tax authorities. The authors used prebuilt data from the Italian Tax Agency’s (Agenzia del Territorio) land registry: to identify ghost buildings hidden from tax authorities, the agency overlaid aerial photographs (representing the state of reality) and digital maps of the land registry. Unlike Casaburi and Troiano (12), who focus on residential land use, we consider potentially unauthorized constructions erected in protected forest areas.

To identify potentially illegal constructions, we refer to the Italian national “Forest Fires Framework Law,” Act no. 353 released on 2000 November 21, which lays out the activities and measures that territorial bodies must undertake to preserve and protect the national forest heritage from fire. In particular, the Forest Fires Framework Law prohibits the construction of infrastructure or buildings for civilian settlements, productive activities, or pastoral and hunting activities in wooded and pasture areas that have experienced fires during the subsequent 10-year period. We use EO data to build a unique dataset of buildings erected in previously burned areas of Sardinia, an Italian region heavily affected by wildfires and illegal construction (13). In order to measure the amount of illegal building activity in the most reliable manner (so as to avoid the inclusion of buildings for which permission was actually granted), we exploit existing national regulations to run robustness tests for our analysis.

We use the wildfire event registrations available in the Sardinia Geoportal (Geoportale Sardegna). The Geoportal provides the georeferenced polygon of each burned area in the region. We focus on municipalities that witnessed at least one fire in protected areas (wood and pastures) between 2005 and 2015. To detect changes in these burned areas, we rely on satellite images provided by the Landsat Program, which represents the longest-running project (1972–present) to collect remote sensing data at an urban scale from satellites. The four-decade images provide a unique resource for studying climate change, the carbon cycle, ecosystems, the water cycle, and changes on the Earth’s surface. The Landsat Program consists of satellites equipped with multispectral sensors that collect imagery with global coverage, providing an important source of data for land use monitoring (14).

We detect suspected changes that have occurred within the burned area polygons on the basis of a multitemporal multispectral index variation. The Landsat-5 and Landsat-8 time series data are processed by the aforementioned automated procedure. Next, we visually inspect the areas that have undergone changes and identify those where an alleged violation has occurred, i.e. a building that appears in a forest or pasture area within a time frame of 1 to 10 years after a wildfire. The polygons where the initial automated procedure detects no change are filtered out from the dataset. Subsequently, the remaining polygons are individually analyzed via visual inspection of very high-resolution images available in the Google Earth Pro catalog to search for constructions that have been built on protected forests and pasture lands.

As a result, we create a unique, municipality-level dataset that provides information on fires and subsequent violations. We then match it with administrative data on local election results. Our application aims to analyze the interplay between mayoral turnover and illegal construction activities to understand how this phenomenon affects voters’ preferences. We also provide a descriptive analysis of the wildfires and their determinants.

Our analysis reveals that surface temperature and rainfall patterns are the key climatic factors influencing the occurrence of fires. This correlation can be attributed to the well-established understanding that wildfires exhibit a pronounced seasonality, as previously investigated by Salis et al. (15). Additionally, our findings indicate that fires tend to spread more extensively in areas with higher altitudes and farther away from urban centers. This result suggests that the challenges faced by local authorities in monitoring and responding to fire incidents are amplified in remote and less accessible areas. We have also observed that burned forest areas are prone to the emergence of illegal constructions. Furthermore, we analyze the relationship between building erected in protected areas and the public’s preferences for the local government representatives. In particular, we find that municipalities with violations in protected areas are more likely to see a change in mayor at the next administrative election; this result is robust to model specifications that include additional factors that might influence mayoral turnover, such as the incumbent’s demographic characteristics (gender, age, and higher education).

Our estimation approach includes municipality fixed effects that account for possible omitted variables that do not change within municipalities over time. These time-invariant variables capture important omitted variables reflecting social and cultural local traits, such as the intrinsic sense of legality of citizens living in a particular municipality and the inherent tendency to weak law enforcement across municipalities resulting in violations of construction bans.

The article is structured as follows. The Results section provides a comprehensive description of the data used in the analysis and presents the key findings. The Discussion section explores the potential policy implications derived from our analysis. The Methods section provides a concise overview of the methodology employed to construct the dataset, along with the empirical strategy. For Supplementary material and information related to the article, we direct the reader to the SI Appendix.

## Results

### Wildfires determinants

This section provides a descriptive analysis of the wildfires phenomenon in Sardinia and its determinants. The analysis is performed at the municipality-month level and our dataset includes all 377 Sardinian municipalities from 2005 to 2015; in this period, 24,358 wildfires were recorded.

**Fig. 1.**
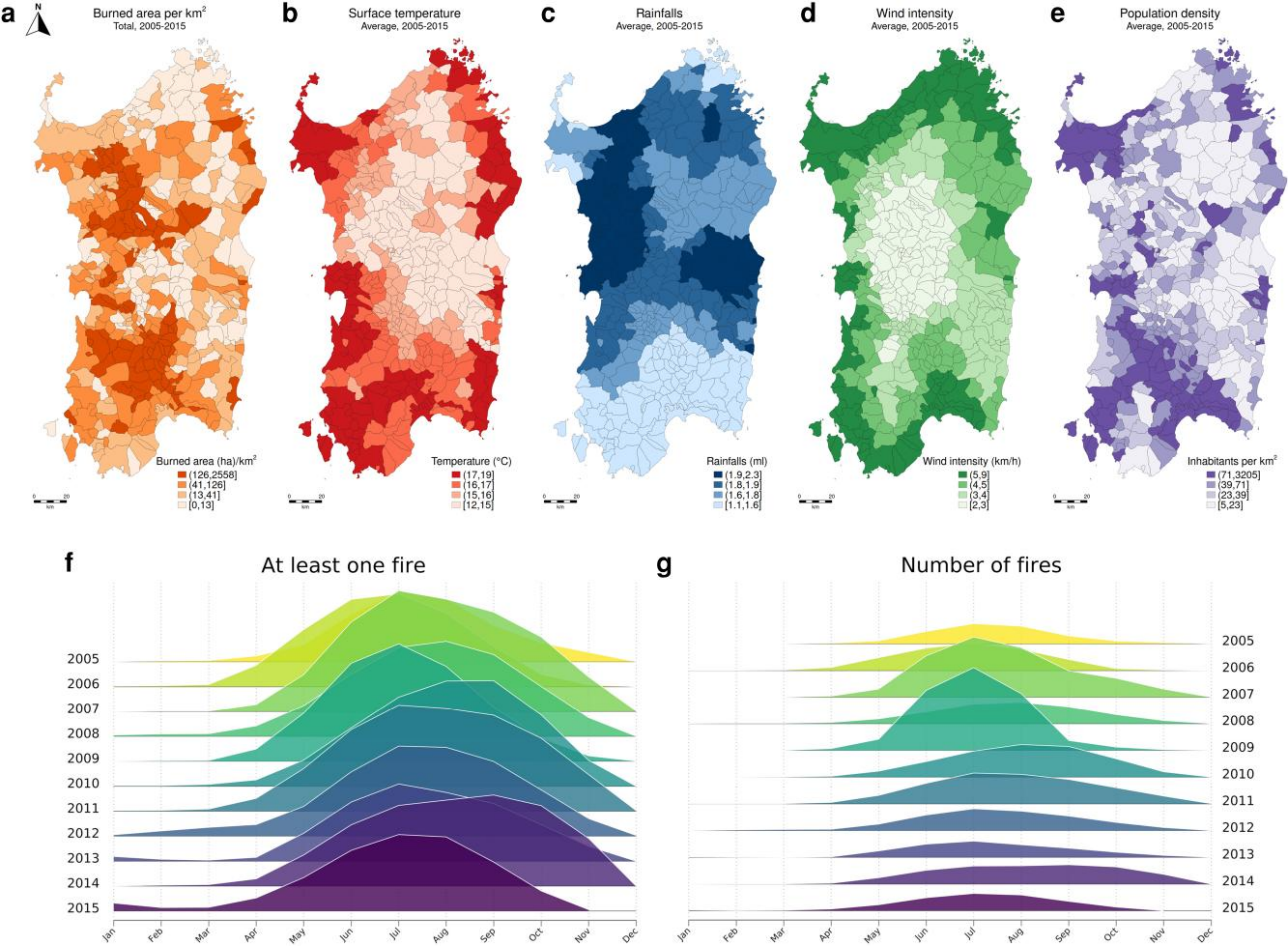
Descriptive statistics of the wildfire phenomenon in Sardinian municipalities and of potentially correlated factors. Map (a) shows the total amount of surface per km^2^ that burned between 2005 and 2015 for each municipality. Maps (b–e) show the average climatic and demographic characteristics of the municipalities; from left to right: surface temperature, rainfalls, wind intensity, and population density. (f) The frequency with which Sardinian municipalities are hit by at least one wildfire in each month is plotted, while (g) shows the number of fires that occurred in each month.

Figure 1 provides a graphical representation of the spatial distribution of the wildfire phenomenon (Fig. 1a) and of potentially correlated factors, i.e. the climatic (Fig. 1b–d) and demographic characteristics of municipalities (Fig. 1e). Moreover, the two joyplots^a^ show the dynamics of wildfires, by plotting in Fig. 1f the frequency of municipalities being hit by at least one wildfire in each month and in Fig. 1g the number of fires that occurred monthly. These graphs highlight the seasonality that characterizes fires, with July often corresponding to a peak (41.8% of municipalities were hit by at least one fire event in July in the years considered, compared to a global mean of 15%). Moreover, they also show how 2007 and 2009 were the years with the largest number of fires, with, respectively, 3,997 and 4,248 events compared to a yearly mean of 1,623.4 events in the years considered.

To complement the graphical analysis, Table 1 provides detailed descriptive statistics of the variables used in the analysis of the determinants of wildfires. The sites hit by wildfires are, on average, 231.86 meters above sea level, 3.02 km from the city center, and 20.96 km from the coast. Rural areas strongly characterize the territory of Sardinia and, as our data show, its population density of around 76.4 inhabitants per km^2^ is among the lowest in Italy.We use a regression analysis to assess the factors that determine the likelihood and extent of wildfires in a municipality. For a robustness check, we perform our analysis using several measures of the wildfire phenomenon and report the results in Table 2. More in detail, firstly, we analyze the probability that municipality *i* is hit by at least one wildfire in month *t* and perform this analysis via a correlated random effects probit model (*Pr(fire)*, first column of Table 2, where average marginal effects are reported). We then analyze, with two-way fixed effects regressions, the amount of municipal surface burned in each month (*Total burned area*, second column of Table 2) and the number of fires (*N. fires*, third column of Table 2). Given that such measurements strictly depend on the size of the municipality, we normalize them with respect to the size of the administrative area (in km^2^). Lastly, we study the determinants of the size of wildfires, normalized with respect to the municipality area (*Avg. burned area*, fourth column of Table 2), again via a two-way fixed effects approach; for this analysis, the dependent variable is the average size of wildfires that occurred in municipality *i* in month *t* and the sample is restricted to municipality-month observations in which at least one wildfire occurred.^b^ The set of covariates includes (i) climatic data of the municipalities where the fires took place (i.e. *surface temperature*, *rainfall* and *wind intensity*) and (ii) the population density for all specifications. Moreover, in the last model, we also account for the geodesic characteristics of the fires, i.e. *altitude* (in log), *distance from city center* (over the size of the municipality in km^2^), and *distance from the sea* (in log). All regressions include time dummies and municipality fixed effects; the latter allow us to account for time-invariant omitted factors, such as socioeconomic and cultural characteristics, as well as the innate propensity for lax law enforcement and monitoring by local institutions.

**Table 1.** Summary statistics of data used for the analysis of wildfires.

Variable	Mean	SD	Min.	Max.	*N*
*Dependent variables*					
At least one fire (dummy)	0.15	0.357	0	1	49,387
Number of fires	0.493	3.839	0	531	49,387
Total burned area (ha)	45.258	975.023	0	140,637.139	49,387
Average burned area per fire (ha)	5.444	26.075	0	1,409.6	7,388
*Climatic data*					
Surface temperature	16.019	7.305	−1.34	32.637	49,387
Rainfall (mL)	1.74	1.396	0.001	10.598	49,387
Wind intensity (km/h)	4.042	2.449	0.02	21.736	49,387
*Municipalities’ characteristics*					
Population density (inhab. per km^2^)	76.414	206.291	4.244	3,259.447	49,387
Municipality area (km^2^)	63.926	61.74	2.474	547.042	49,387
*Wildfires’ characteristics*					
Avg. altitude (m.a.s.l.)	231.857	220.772	0	1,556	7,388
Distance from city center (km)	3.015	1.622	0.022	12.029	7,388
Distance from the sea (km)	20.962	14.106	0.045	52.65	7,388

Dependent variables and Climatic data are referred to municipality *i* during month *t*. Wildfires’ characteristics and Average burned area per fire, average characteristics of the wildfires that hit a municipality in a given month; the sample is restricted to the municipality-month observations in which at least one wildfire occurred; m.a.s.l., meters above sea level; ha, hectares.

**Table 2.** Determinants of the wildfire phenomenon.

	Pr(fire)	Total burned area/ km^2^	N. fires/ km^2^	Avg. burned area/ km^2^
Surface temperature	0.016***	0.891***	0.004***	−0.001
	(0.003)	(0.165)	(0.001)	(0.014)
Rainfall	−0.012***	−0.216	−0.002**	−0.010
	(0.004)	(0.156)	(0.001)	(0.018)
Wind intensity	0.006***	0.107	0.0003	−0.001
	(0.002)	(0.071)	(0.000)	(0.007)
Population density (log)	0.007	0.310	0.023*	−0.226
	(0.050)	(2.558)	(0.013)	(0.210)
Altitude (log)				0.039***
				(0.012)
Distance from city center (norm.)				1.041***
				(0.237)
Distance from the sea (log)				0.005
				(0.021)
Observations	41,470	49,387	49,387	7,388
Municipality FE	YES	YES	YES	YES
Time FE	YES	YES	YES	YES
Number of municipalities	377	377	377	375

Standard errors in parentheses: ***$P<0.01{,}^{**}P<0.05{,}^{*}P<0.1$

The dependent variable in *Pr(fire)* is a dummy variable equal to 1 if municipality *i* experienced at least one fire in month *t* and 0 otherwise; average marginal effects from a correlated random effects probit model are reported, with municipality fixed effects proxied by mean values of the time-varying covariates. The dependent variables in *Total burned area/* km^2^ and *N. fires/* km^2^ are, respectively, the sum of the size of all wildfires and the number of wildfires that hit municipality *i* in month *t* over the municipality area (km^2^). The dependent variable in *Avg. burned area/* km^2^ is the average size of the fires that hit municipality *i* in month *t* over the municipality area (km^2^) and the sample is restricted to municipality-month observations in which at least one wildfire occurred (for which we also have average geodesic characteristics, i.e. altitude, distance from the sea and the city center); altitude and distance from the sea are normalized using a logarithmic transformation while the distance from the city center (in km) is normalized with respect to the municipality area (km^2^). All models include municipality and time fixed effects.

Our findings indicate that higher surface temperatures increase the likelihood that a municipality will be affected by at least one wildfire. Consequently, this leads to an increase in both the average size of the burned area and the overall number of fires. In addition, rainfall has a mitigating effect, acting in the opposite direction by reducing the occurrence of fires. Consistently with previous research, wind intensity increases the probability of a wildfire igniting. Furthermore, we find that the number of fires per km^2^ that monthly hit Sardinian municipalities is positively, although weakly, correlated with population density. Finally, the average size of the wildfires (fourth column of Table 2) is positively correlated with both the altitude of the wildfire-burned area and its distance from the city center.

### Buildings in protected areas and mayoral turnover

The data on wildfires are the starting point for our study on the detection of illegal buildings. Firstly, we restrict our attention to the fires that led to the construction ban^c^ and then, using the methodology described in the Method section, we screen satellite and aerial images for up to 10 years after the fire and search for new building constructions. If the latter are built at least 1 year after the fire event, that is, not in the same year as the fire, they may be considered potentially illegal.^d^ This criterion is applicable for up to 10 years post-fire since, after this period, the area is no longer considered protected. Therefore, the resulting data on alleged violations cover the years 2006 to 2019; we refrained from considering later years to avoid possible confounding effects of the COVID-19 pandemic on constructions.

**Fig. 2.**
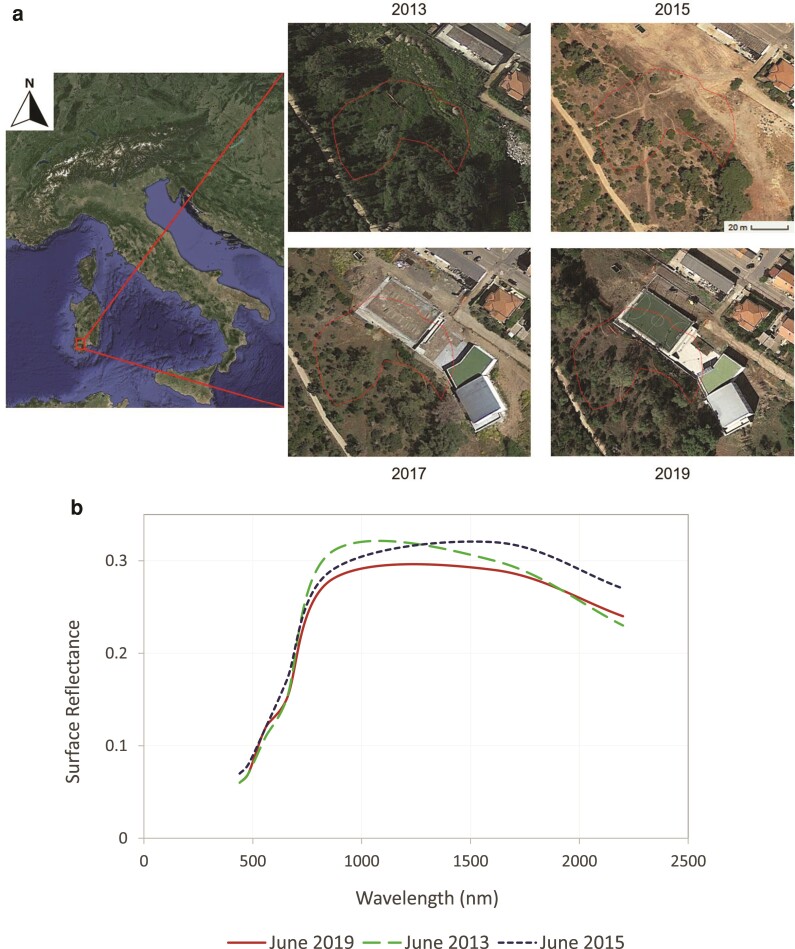
Example of a potential violation after a wildfire. Images in panel a (Credits: Google Earth Pro) show the land cover evolution of an area where a wildfire occurred in 2014 (highlighted polygon), from 2013 (before the fire) to 2019 (alleged violation). Graph in panel b shows the surface reflectance acquired from satellite averaged within the aforementioned polygon hit by a fire. In particular, panel b reports the reflectance derived from three Landsat-8 images: before the fire (7 June 2013, dashed line), after the fire but before the construction (29 June 2015, long dotted line), and, finally, after a building was constructed (24 June 2019, solid line).

Figure 2 provides a representative example of a potential violation. The red border polygon represents an area labeled as “forest” land by the Sardinia Region Geoportale, where a wildfire occurred in 2014. Therefore, any construction within its boundaries should have been prohibited for 10 years. (a) The same area before the fire (2013), after the fire (2015), and finally with a structure built in 2019, using earth observation data provided by Google Earth Pro is shown. At the same time, (b) the corresponding variation in terms of surface reflectance acquired from space, i.e. the fraction of electromagnetic energy reflected by a surface with respect to the total (solar) incident energy to that surface is shown. (b) The trend of the surface reflectance obtained from Landsat-8 images in June 2013 (dashed line), 2015 (long dotted line), and 2019 (solid line) is reported. Before the construction, the change in land cover due to the 2014 fire corresponds to a shift in the peak of the reflectance, moving from the near-infrared band (associated with high vegetation density) to the short-wave infrared band (associated with low vegetation density). Moreover, as expected, the surface reflectance in the same electromagnetic spectrum range (particularly across the visible and short-wave infrared) differs from what was recorded in the same area before the potential violation. The surface reflectance trend was obtained by averaging the values of the pixels from the Landsat-8 images that fall within the burned polygon or are intersected by the (red) edge of the polygon.

**Fig. 3.**
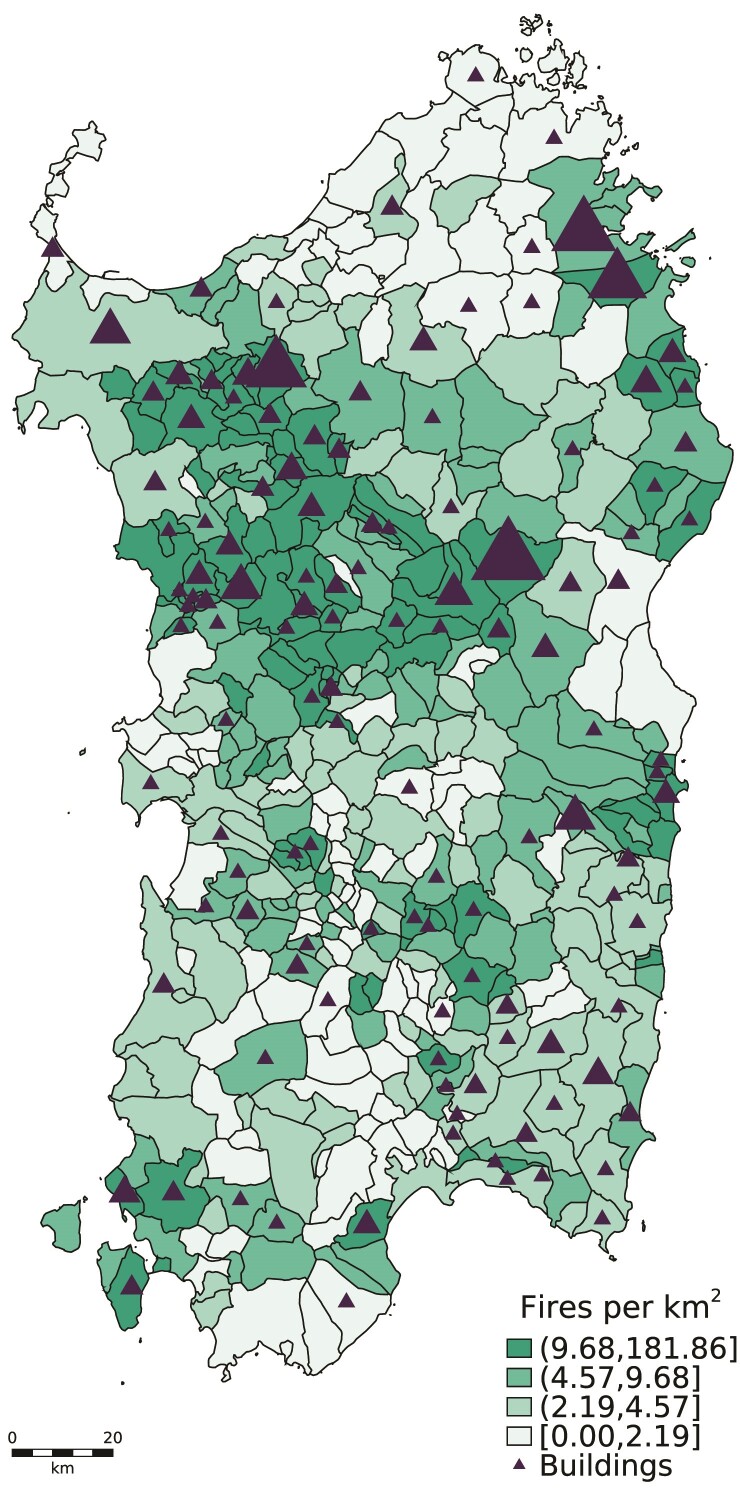
Map of wildfires in protected areas and detected buildings. The intensity of the color of each municipality indicates the number of wildfires that occurred in the period 2005–2015. The presence of a triangle signals a municipality where buildings in protected burned areas were detected and confirmed by visual inspection in the period 2006–2019; the size of the triangle is proportional to the number of detected buildings.

The results of our building detection methodology are presented in Figure 3. The intensity of the color of each Sardinian municipality is proportional to the number of fires in protected areas that occurred in the period 2005–2015. The triangles identify municipalities in which at least one alleged violation was observed in the period 2006–2019; the size of each triangle is proportional to the number of buildings detected. More frequent wildfire events are observed in the northwestern province of Sassari and in the southern province of Cagliari. It is also interesting to highlight that potentially illegal building activity affects the region quite homogeneously.

Using our data on buildings erected in protected areas, we next focus on testing whether their presence affects mayoral re-election prospects. The time frame covered in this part of the analysis is the same as the one studied for building violations, i.e. from 2006 to 2019, to which we combine data on local election outcomes. Our dependent variable of interest is whether there is a change of mayor after local elections. Therefore, the data refer to election years only.

Of the 989 electoral events, 67.1% correspond to cases in which municipalities experienced a change of mayor. Mayors are men in 89.8% of the cases, with an average age of 52.1 years, and 45.1% of them have a higher education. Regarding potential violations, 32.6% of the municipalities had at least one building erected in protected areas during the period considered; overall, we have observed 283 potential violations. Lastly, the average income per capita in the sample considered is €7,299. Descriptive statistics of the variables used for this part of the analysis are reported in the Table S.1.

We use a two-way fixed effects linear probability model, where the dependent variable of interest is a dummy indicating a change in the mayor in the *i*th municipality in the year *t* when local elections took place (the variable equals one if the outgoing mayor is not re-elected, irrespective of whether they ran again). Our main independent variable of interest is a variable indicating the total number of buildings detected in protected areas of municipality *i* in the years up to t−1. After testing for the presence of this association in the simplest model (Model (1)), where we only use potential violations as the independent variable, we include additional factors that might influence mayoral turnover and check whether the positive association we found still exists. In particular, we account for the characteristics of the incumbent mayors first, i.e. gender, age, and whether s/he has a Bachelor’s degree or higher (Model (2)). Lastly, we also include among the regressors the economic characteristics of the municipality, i.e. income per capita lagged one period—to avoid reverse causality concerns (Model (3)). All models include both municipality and year fixed effects; municipality fixed effects also capture the intensity of the wildfire phenomenon in each specific city, which in turn affects the likelihood of illegal constructions being built. Results are reported in Table 3.

**Table 3.** Determinants of mayoral turnover.

	Mayoral turnover		
	*(1)*	*(2)*	*(3)*
Buildings in previous years	0.039**	0.050***	0.047***
	(0.019)	(0.016)	(0.017)
Mayor gender (male)		0.021	0.020
		(0.073)	(0.074)
Mayor age		0.019***	0.019***
		(0.003)	(0.003)
Mayor higher education		0.048	0.051
		(0.048)	(0.048)
Income per capita (log, t−1)			−0.337
			(0.451)
Observations	989	878	878
Municipality FE	YES	YES	YES
Year FE	YES	YES	YES
Number of municipalities	377	368	368

Robust standard errors in parentheses: ***$P<0.01{,}^{**}P<0.05{,}^{*}P<0.1$

The dependent variable for all model specifications is a dummy variable equal to 1 if municipality *i* had a new mayor elected in year *t* and 0 otherwise. The sample is restricted to election years only. The covariate *Buildings in previous years* is a count variable equal to the number of potential violations detected in protected areas of municipality *i* in the years up to t−1.

Our results show that there is a positive association between the presence of potentially illegal buildings in the years preceding the local elections and the probability of mayoral turnover. Furthermore, this effect is robust to model specifications that include additional regressors, such as the incumbent mayor’s demographic characteristics and income per capita. Although the latter does not seem to affect mayoral turnover, we find that age is positively correlated with the probability of a change in mayoral leadership, i.e. older mayors are less likely to be re-elected. We do not find any association with gender or the higher level of education of mayors.

To limit the possibility of including buildings that were granted a construction permit prior to the fire, we provide estimates based on alternative inclusion criteria of buildings in our dataset (Table S.2). In particular, we test whether our results hold when we disregard buildings that were erected 2 to 4 years after the fire.^e^ Our results are robust to these tests.

## Discussion

The high number of wildfires over the past decade has been the result of several factors, including climate and poor land monitoring by local authorities (16, 17). We find a strong correlation between the probability of wildfires, as well as the intensity of the phenomenon (measured by the total burned area and the number of fires), and climatic conditions, particularly surface temperature and inadequate rainfall. In addition, wind intensity has been identified as a contributing factor to fire ignition, as previously emphasized in existing literature (see, among others, (18–20)). We also observe that the phenomenon reaches its peak during the summer months, particularly in July. These results are consistent with the work of Salis et al. (15), who study the seasonal patterns of wildfire exposure in Sardinia and find that the period from July 16 to August 15 has the highest probability of wildfire events.

Our analysis also highlights that, once a wildfire ignites, its size depends on the specific characteristics of the location. In particular, a higher surface temperature is associated with a higher probability that a municipality will face wildfires and, consequently, is also associated with a larger total burned area and a higher number of fire events. In contrast, rainfall has the opposite effect, mitigating the impact of wildfires. Furthermore, the size of wildfires is related to the geographical characteristics of the location, with larger fires spreading in high-elevation areas and further away from the city center. This result can be explained in two ways. On the one hand, areas farther away from the city center are more likely to be characterized by more vegetation, such as forests and fields, therefore being more prone to fires, especially during the summer season. On the other hand, we interpret this result as a sign that intervention is more difficult in areas where access is limited. In addition, the peripheral areas of the municipalities are also more likely to serve as sites for illicit activities, such as the constructions we consider in our study. The main policy implications for local authorities are, therefore, to better control these areas to preserve and prevent violations of the natural landscape. It is also interesting to stress that we find a positive, albeit weak, relationship between the number of wildfires and population density. This result supports field evidence from the wildfire research literature, which highlights that areas with higher population density have more road and infrastructure networks, and therefore more human activity, which can influence the prevalence of wildland-urban interface, ultimately leading to an increase in the number of wildfires (see Refs. (21–24)).

There is a sizable body of research on retrospective voting at a national level (25). However, our understanding of how accountability operates for local governments in addressing illegal activities perpetrated within their municipality remains limited. We use EO data to measure a specific form of illegal behavior that concerns inhabitants, i.e. illegal construction activity, and explore the possible links with the re-election of local officials. This is a significant gap that our work addresses, as most elected officials work at a local level, where their decisions have a more direct and immediate impact on individuals’ daily lives than those made at a state or regional level.

In the analysis of the electoral consequences of building activities in protected areas, we observe a positive association between constructions built in protected areas and mayoral turnover. We interpret this effect as evidence that voters punish incumbent mayors in the ballot box: the presence of violations in the years preceding the local elections makes it more likely that the mayor will change. Several potential mechanisms can support this interpretation. On the one hand, illegal constructions in protected areas cause an economic loss that should be prevented by local authorities: these buildings are, in fact, outside the formal property market and are not part of the tax base, which results in significant losses and distortions in government revenues (26). On the other hand, the presence of buildings in protected areas can be a proxy for wider phenomena at the municipal level that cause citizens to lose trust in the incumbent administration. For example, unregulated building works contribute to landscape degradation, a form of environmental loss punished by voters. Another example is related to the fact that illegal buildings are often associated with a lack of surveillance or law enforcement at different levels, all the way up to individual bureaucrats, which may even lead to their own personal gain. Private gains may include not only direct material benefits for officials, such as money, but also indirect benefits, such as improved prospects for re-election (27, 28). In these cases, it is not the mere presence of unauthorized buildings that the voters are punishing, but more the general attitude of the local authorities. Our study reveals that elections serve as a means to hold public officials responsible for their local governance and to punish them (29, 30). In other words, mayors who failed to prevent illegal constructions during their time in office are less likely to be re-elected.

This paper sheds light on the consequences of the increasing number of wildfires that have drastically altered the landscapes of various countries. In our specific case study, this has resulted in the construction of new buildings in forest and pasture areas that have been ravaged by fire. Our research methodology can be applied to both developing and developed economies, especially when there are regulations in place to control building work in fire-affected areas. The proposed approach allows us to gain a more thorough understanding of the wildfire phenomenon in areas where there is a subsequent interest in carrying out construction work that illegally changes land use. For instance, in the United States, housing growth has markedly changed protected areas and their surroundings (31), and wildfire incidence is expected to worsen these changes. The growth of illegal constructions in protected areas should be quantified to inform local authorities and assess the conservation threats posed by these unlawful activities. Ultimately, local politicians who protect the landscape of their municipality will be politically rewarded by a broader public consensus.

In summary, EO data are a powerful and objective tool to evaluate the effectiveness of governance in a specific area. Local authorities are, in fact, responsible for regulating and enforcing building laws, while national or regional governments oversee the relevant legislation. Both areas require an accountable system to ensure that laws and regulations are respected and enforced. Efforts to combat illegal building require accurate information regarding the presence of violations, particularly in remote and hard-to-access areas located far from urban hubs. This entails comparing the actual situation on the ground with the legal requirements and regulations in place. As emphasized in our study, it is crucial to verify the on-site findings to ensure their reliability. The proposed procedure provides accurate information to the relevant authorities, including local government officials and law enforcement agencies. Local authorities can use this evidence to address the problem of illegal construction in areas that have been severely affected by forest fires. They can employ a range of measures, such as ensuring efficient and timely enforcement of building regulations, maintaining vigilant monitoring of affected areas, and actively raising public awareness about this issue.

## Methods

The first part of this section presents a brief description of the methodology through which we detect buildings in previously burned areas and describes how the resulting dataset is combined with climatic, electoral, and demographic data. More detailed information on the approach implemented to generate our dataset using Earth observation data can be found in the SI Appendix, Section B. The second part of this section describes the econometric approach employed to analyze the determinants of wildfires and the association between violations and mayoral turnover.

### Dataset construction

#### Wildfire and violation data

As mentioned above, our legal framework is the National Law Act no. 353/2000 (called the “Framework Law on Forest Fires”). In particular, the first subparagraph of Article 10 states: “On Wooded areas and pastures whose soils have been crossed by fire […] the construction of buildings, as well as the construction of structures and infrastructures intended for civil settlements and production activities *are also prohibited for 10 years*, except for cases where the authorization or concession that permits the above-mentioned constructions has been granted prior to the fire event, and considering the development plan in force on that date.” This piece of legislation is straightforward, without all the exceptions that usually coexist with regulations, and it clearly defines the range of action. In compliance with the Framework Law on Forest Fires, each detected wildfire in forest and pasture areas was subsequently monitored for 10 years, whenever feasible, until 2019. We limit the monitoring activity to 2019 to avoid possible confounding effects of the COVID-19 pandemic and the consequent limitations on mobility and economic activities, which started in Italy at the beginning of 2020 and consequently may have had an impact on illegal building activity. Furthermore, to avoid the inclusion of buildings that had received a permit prior to the fire, we only consider constructions that appear in the year after the fire or later. We also provide robustness checks for our analysis for different inclusion criteria of buildings, i.e. erected 2, 3, or 4 years after the fire, to avoid the inclusion of buildings for which an extension was granted.

We chose the region of Sardinia, the second-largest island in the Mediterranean Sea, as our case study because, out of all the regions in Italy, it is the one that is most affected by land consumption due to fires. These data are confirmed by the European Forest Fire Information System database (EFFIS, 2020), which provide detailed and updated data on wildland fires in Europe (https://effis.jrc.ec.europa.eu) and that even with coarse spatial resolution, clearly indicates that Sardinia, together with Sicily, have been the most affected regions in recent years. We have also chosen Sardinia as our case study because the regional offices that govern its entire territory provide an efficient and complete database of fires, comprehensive of every wildfire that has ignited across the island on a yearly basis since 2005.

Landsat products are freely available on the U.S. Geological Survey (USGS) archive, which is America’s largest water, earth, and biological science and civilian mapping agency (https://earthexplorer.usgs.gov/, last accessed on 19 October 2023). Until 2011, Landsat-5 was the main imagery acquisition tool used for our dataset; however, starting in 2013, Landsat-8 became the primary source due to its enhanced capabilities for long-term, high-frequency monitoring applications. We did not consider Landsat-7 images for the dataset generation, given the data gaps in the products collected after 2003 May 31, due to the failure of the scan line corrector. Each Landsat satellite is in a near-polar, sun-synchronous orbit and provides complete coverage of the Earth every 16 days. We exploited these satellite images to develop an objective procedure for detecting suspected land cover changes based on the temporal variation of two optical indexes, which assume values that can be representative of different land cover types (e.g. urban, vegetation). We focused on municipalities that witnessed at least one wildfire from 2005 to 2015. We had to handle temporal gaps in the data due to limitations in satellite data availability in specific time periods. Indeed, despite the frequent repeats of Earth coverage, satellite optical remote sensing suffers from meteorological stochasticity and, for our purposes, cells with clouds simply translate into missing values. Thus, we had to cope with satellite product gaps for 2008 and 2010, mainly due to a high number of cloudy images and acquisition issues, which did not allow the collection of a sufficient number of Landsat-5 images for the algorithm to be applied. Furthermore, in 2012 there were no Landsat-5 data available for Sardinia, as the mission suffered fluctuations in its performance and was subjected to various mechanical failures starting in late 2011. The SI Appendix, Section B.1, reports additional details on the satellite products considered to generate the dataset.

For the purposes of this work, we have implemented an algorithm that allows us to filter out burned areas where no change occurred in the 10 years after a wildfire, for the period 2005–2015. The algorithm compares the yearly variation of two optical indexes computed within the polygon of each burned area with the values obtained in its surroundings. If the difference did not exceed an empirically defined threshold for both indexes, the polygon was filtered out of the dataset, assessing that no change occurred during the period considered. The procedure is based on the computation of the Normalized Difference Built-up Index (NDBI) and the Surface Reflectance-derived Normalized Difference Vegetation Index (NDVI). The latter index is used to quantify vegetation greenness and is useful in understanding vegetation density and assessing changes in plant health (32). In fact, green, healthy vegetation reflects solar radiation in the near-infrared (NIR) region, while it absorbs red light in the visible part (RED) of the electromagnetic spectrum in the presence of photosynthetic activity, in which there is absorption by chlorophyll (33). NDVI is computed by exploiting the reflectance in the red (636–673 nm) and in the near-infrared (772–879 nm) spectral bands, as shown in the following formula:


$$\mathrm{NDVI}=\left(\frac{\mathrm{NIR}-\mathrm{RED}}{\mathrm{NIR}+\mathrm{RED}}\right).$$

The Normalized Difference Built-up Index highlights urban areas with higher reflectance in the short-wave infrared (SWIR) region, compared to the near-infrared (NIR) region to highlight built-up areas. It is ratio-based to mitigate the effects of terrain illumination differences, as well as atmospheric effects (34, 35). NDBI is computed on the basis of the reflectance in the NIR and SWIR (1,547–1,651 nm) bands of the electromagnetic spectrum, according to the following formula:


$$\mathrm{NDBI}=\left(\frac{\mathrm{SWIR}-\mathrm{NIR}}{\mathrm{SWIR}+\mathrm{NIR}}\right).$$

The alerts generated by the algorithm in the first step aimed to limit the analysis to areas and years where a possible change, that is, a suspected violation, occurred. This significantly helps us to restrict our sample since the next stage of the dataset generation process was based on visual inspection, a highly time-consuming process. Visual inspection, on the other hand, can provide reliable and validated data on illegal buildings, which is the foundation of this study. The areas where a possible change may have occurred were visually inspected using very high-resolution historical images available in the open software Google Earth Pro for the period 2005–2019. This activity was crucial in assessing whether each change was a building erected in the burned area. Additional details on the change detection procedure, its validation and the visual inspection phase are provided in the SI Appendix, Sections B.2 and B.3.

Data on wildfires are then aggregated at the municipality-month level, covering the period 2005–2015. This dataset includes the number of fires, the total burned area, and the average burned area per fire. We also gather information on the geodesic characteristics of the wildfires, as described below.

Data on potential violations are aggregated at the municipality-year level, as the availability of satellite images does not allow us to assess the specific month in which the construction of a building starts or ends. Data cover the period 2006–2019 and consist of the number of buildings in protected areas detected for every municipality in every year, excluding those for which satellite data were unavailable (i.e. 2008, 2010, and 2012). For the missing years of satellite image acquisition, buildings were attributed to the subsequent year with available data (i.e. 2009, 2011, and 2013).

#### Geodesic, population, and electoral data

We complement our datasets on wildfires and potential violations with data regarding the geographic characteristics of the burned areas and with population and electoral data.

In the analysis of the wildfire determinants, we consider the altitude of the burned area provided by the Shuttle Radar Topography Mission (SRTM) and available on the Earth Explorer USGS web portal (36). The burned area is identified via its centroid, as well as its (geodesic) distance from the coastline and the city center (which we identify as the centroid of the municipality’s administrative area). Distances are computed according to the minimal-distance criterion from the centroid of the burned area. With the inclusion of such geographic information, we aim to control how easily local authorities can monitor the territory, under the hypothesis that areas far away from residential areas and the city center are harder to monitor. To control for municipality-specific characteristics, we retrieved the population density from the Italian National Statistical Office (ISTAT) and per capita income from the Italian Home Office (Ministero dell’Interno). In particular, we include population density in the analysis of the determinants of wildfires to check whether their causes are mostly climatic or human related.

For our analysis of mayoral turnover, we have collected data on mayors and their personal characteristics (age, gender, and education level) from the Registry of Local Administrators provided by the Italian Home Office for the years 2005 to 2019.

#### Climatic data

We gather climatic variables from the Copernicus Program databases. Copernicus is the European Earth Observation program: it started in 2014 and follows the GMES, the Global Monitoring for Environment and Security initiative. The Copernicus Program is a public framework that allows full, free, and open access to all environmental monitoring data collected in the European Union.

Among the six different thematic information services currently active within Copernicus, for our analysis we use the Copernicus Climate Change Monitoring Service (C3S), which produces data related to climate changes. Specifically, we use the ERA5-Land dataset (10.24381/cds.e2161bac, last accessed on 19 October 2023) that consists of climate variables obtained from a combination of surface-level observations recorded by sensors on the ground and data provided by the H-TESSEL land surface model. From this database, we extract a set of climatic data on wind, air temperature, and total precipitation:

- *Wind intensity* (km/h) is calculated by combining the eastward and northward wind components, measured in meters per second at a height of 10 meters above the Earth’s surface. The resultant vector represents the speed and direction of the horizontal wind at 10 meters above the ground;
- *Surface temperature* is measured in Kelvin and is converted to degrees Celsius (°C) by subtracting 273.15;
- *Rainfall* is measured in milliliters (mL) of liquid water equivalent over the preceding time interval.

The climatic variables, provided with a spatial coverage of 0.1×0.1 longitude/latitude degree, were later processed and combined to analyze monthly information at the municipal level, which is the smallest geographic unit in Italy.

### Econometric strategy

We study the determinants of the wildfire phenomenon via a two-way fixed effects regression:


$$w_{i,t}=x_{i,t}\beta +\alpha_{i}+\tau_{t}+u_{i,t}\;i=1,\ldots ,377;\;t=2005m1,\ldots ,2015m12,$$

where $w_{i,t}$ is an index of whether and how severely municipality *i* was hit by wildfires during month *t*, $x_{i,t}$ is a vector of covariates, $\alpha_{i}$ is an unobservable municipality fixed effect and $\tau_{t}$ is a set of time dummies. When we estimate the probability that a municipality is hit by at least one fire event in a given month, we resort to a correlated random effects probit approach, where fixed effects are proxied by the municipality-level averages of the time-varying covariates (see Refs. (37–39)).

The association between buildings erected in protected areas and outcomes at local elections is analyzed via a two-way fixed effects linear probability model of the form:


$$\begin{array}{rl} & y_{i,t}=\lambda b_{i,t-1}+x_{i,t}\beta +\alpha_{i}+\tau_{t}+u_{i,t}\\ & \;i=1,\ldots ,377;\;t=2006,\ldots ,2019, \end{array}$$

where $y_{i,t}$ is a dummy indicating mayoral turnover in the *i*-th municipality in the year when local elections took place, *t* (the variable equals 1 if the outgoing mayor is not re-elected, irrespective of whether they ran again). Our independent variable of interest, $b_{i,t-1}$, is a count variable indicating the number of buildings erected in protected areas of municipality *i* in the years up to t−1. The set of covariates, $x_{i,t}$, includes both the characteristics of the incumbent mayors, i.e. gender, age, and level of education (i.e. a dummy indicating whether the incumbent mayor has a Bachelor’s degree or higher), and the economic characteristics of the municipality, i.e. income per capita (lagged one period, to avoid reverse causality concerns). All models presented include municipality fixed effects, $\alpha_{i}$, and year dummies, $\tau_{t}$.

## Supplementary Material

pgae466_Supplementary_Data

## Data Availability

Data on wildfires are available in the repository of Geoportale Sardegna (https://www.sardegnageoportale.it/). Shuttle Radar Topography Mission (SRTM) dataset, from which we gather the geodesic characteristics of the burned areas, and Landsat satellite data are available in the Earth Explorer USGS repository (https://earthexplorer.usgs.gov/, last accessed on 2023 October 19). Climatic data are available in the repository of the Copernicus Climate Change Service, via the Copernicus Climate Data Store (https://cds.climate.copernicus.eu, last accessed on 19 October 2023). Data on population density and municipality area are available in the repository of the Italian National Statistical Institute (ISTAT, https://www.istat.it). Data on local election outcomes are available in the repository of the Italian Home Office (Ministero dell’Interno, https://dait.interno.gov.it/elezioni/anagrafe-amministratori). The code and data for the replication of the analyses are available at https://doi.org/10.17605/OSF.IO/KV7TR. Additional data and materials are included in the SI Appendix.

## Notes

a Joyplots were created using the Stata command joyplot (40).

b The number of municipalities included in the last model decreases to 375 as two of them, Belvi and Sant’Antonio Di Gallura, were never hit by a fire in the years considered.

c Such areas are those labeled as “forest” or “pasture,” according to our main legislative reference (“Forest Fires Framework Law,” Act no. 353 of 2000 November 21). Of the total of 24,358 fire events included in our dataset, 10,843 (44.5%) occurred in such areas, which consequently became a protected area.

d The Decree of the President of the Republic no. 380/2001 requires the construction work to begin within 1 year from the building permit being issued; the building must be completed within 3 years unless a 1-year extension has been granted. By excluding buildings that appear the same year as the fire, we restrict our analysis to constructions that are more likely to have been built in violation of regulations. An extraordinary extension (up to 2 years) was introduced with Decree no. 69/2013. Based on this, we also provide robustness tests for our analysis, as explained later.

e The aforementioned Italian legislation (Decree no. 380/2001) requires construction work to begin within 1 year of the permit being issued and end within three. Extensions of 2 years were possible during the observation period (Decree no. 69/2013).
