# Supplementary material for: New metrics for governance in the era of earth observation data: Monitoring violations after wildfires
Source: PNAS Nexus. 2024 Oct 16;3(11):pgae466. doi: 10.1093/pnasnexus/pgae466 (PMC11601981; doi:10.1093/pnasnexus/pgae466)
Supplement: pgae466_Supplementary_Data [file pgae466_supplementary_data.pdf]

Supplementary Information for

New Metrics for Governance in the Era of Earth Observation Data: Monitoring Violations after Wildfires

Germana Corrado[1], Luisa Corrado[2,*], Fabio Del Frate[3], Davide De Santis[3], and Francesca Marazzi[2]

[1]Department of Management and Law, University of Rome“Tor Vergata”, 00133 Rome, Italy
[2]Department of Economics and Finance, University of Rome “Tor Vergata”, 00133 Rome, Italy
[3]Department of Civil and Computer Science Engineering, University of Rome “Tor Vergata”, 00133 Rome, Italy

This PDF file includes:

Tables S1 to S6
Supplementary text including methodology appendix

A Results: Additional tables

Table S.1. Summary statistics the data used in the analysis of mayoral turnover

Variable	Mean	Std. Dev.	Min.	Max.	N
Dependent variables					
Change of mayor	0.671	0.47	0	1	989
Municipality-level covariates					
Buildings in previous years	0.299	1.168	0	16	989
Income per capita ($t-1$, €)	7299.528	1685.635	3153.318	15768.4	989
Incumbent mayor's demographic characteristics					
Mayor gender (male)	0.898	0.302	0	1	916
Mayor age	52.066	9.865	27	78	915
Mayor higher education	0.451	0.498	0	1	879

The sample is restricted to election years only. *Buildings in previous years* is a count variable corresponding to the number of buildings erected in protected areas in municipality i in the years up to $t-1$ (with election year excluded). *Demographic characteristics* refer to the mayor in office before the elections.

Table S.2. Determinants of mayoral turnover, robustness test on buildings erected in protected areas

	Mayoral turnover									
		2 years after fire			3 years after fire			4 years after fire		
	(3)	*(1a)*	*(2a)*	*(3a)*	*(1b)*	*(2b)*	*(3b)*	*(1c)*	*(2c)*	*(3c)*
Buildings in previous years	0.0472***									
	(0.0169)									
Buildings in previous years (+2 yr)		0.0380**	0.0492***	0.0464***						
		(0.0192)	(0.0161)	(0.0170)						
Buildings in previous years (+3 yr)					0.0388*	0.0559***	0.0528**			
					(0.0233)	(0.0212)	(0.0219)			
Buildings in previous years (+4 yr)								0.0437*	0.0629***	0.0598**
								(0.0247)	(0.0226)	(0.0234)
Mayor gender (male)	0.0205		0.0202	0.0197		0.0206	0.0201		0.0212	0.0206
	(0.0736)		(0.0735)	(0.0737)		(0.0735)	(0.0737)		(0.0735)	(0.0736)
Mayor age	0.0189***		0.0189***	0.0188***		0.0189***	0.0189***		0.0189***	0.0189***
	(0.00256)		(0.00256)	(0.00256)		(0.00256)	(0.00256)		(0.00255)	(0.00255)
Mayor higher education	0.0506		0.0472	0.0502		0.0463	0.0494		0.0457	0.0488
	(0.0485)		(0.0481)	(0.0485)		(0.0481)	(0.0485)		(0.0481)	(0.0485)
Income per capita (log, $t-1$)	−0.337			−0.347			−0.367			−0.358
	(0.451)			(0.449)			(0.447)			(0.448)
Observations	878	989	878	878	989	878	878	989	878	878
Municipality FE	YES	YES	YES	YES	YES	YES	YES	YES	YES	
Year dummies	YES	YES	YES	YES	YES	YES	YES	YES	YES	
Number of municipalities	368	377	368	368	377	368	368	377	368	368

Robust standard errors in parentheses: *** p<0.01, ** p<0.05, * p<0.1

The dependent variable for all model specifications is a dummy variable equal to 1 if municipality i had a new mayor elected in year t and 0 otherwise. The sample is restricted to election years only. The covariates *Buildings in previous years (x yr)* are the same as the original variable *Buildings in previous years*, but where constructions built x years after the fire are disregarded. Refer to Table 3 in the main text for a description of the covariates. Model *(3)* with the original model is reported for references.

B Methodological appendix

B.1 Satellite data

We used Landsat-5 imagery from 2005 to 2011 and Landsat-8 imagery from 2013 onward to select the polygons for the visual inspection procedure to detect violations. We did not consider Landsat-7 images for the dataset generation, given the data gaps in the products collected after May 31, 2003, due to the failure of the scan line corrector. We selected five Landsat images for each year and used them to create the Sardinia "mosaic" to obtain a yearly picture of the entire region of interest. Table S.3 lists the satellite products used, while Tables S.4 and S.5 report the characteristics of Landsat 5 and Landsat 8, respectively.

Almost all of the satellite images considered were acquired on a date selected from the July-August period to minimize possible seasonal effects on the calculated indexes and, at the same time, increase the chances of selecting cloud-free products from those available in the Landsat data catalog.

B.2 Land cover changes screening procedure

As this study focuses on acquiring sound, validated data on illegal buildings, we conducted a visual inspection of very high-resolution satellite and aerial imagery available as historical images in Google Earth Pro (see section **B.3**). Although this technique can be considered one of the most accurate in Earth Observation data exploitation, it requires prohibitive processing time, particularly when dealing with datasets on the order of 10^5 or more, as in our case. To reduce the time-consuming process of manually inspecting each burned polygon over a ten-year period following an event, we have implemented an automated procedure. Considering all fires reported between 2005 and 2015, we filter out burned areas smaller than 10 hectares where no change has occurred. We exploited the data provided by Sardinia Geoportal, consisting of vector tiles showing the burned areas for each year considered (in shapefile format, i.e., a vector data format for geographic information system (GIS) files, specifically used to encode geographic information). In the proposed first step of the methodology, we overlap the Landsat satellite images with all the vector tiles to:

- monitor the burned areas for ten years after the fire event and identify possible yearly changes within those polygons over time. This method makes use of the spectral response of changes in land cover in terms of the surface reflectance data obtained by satellite;
- separate the polygons into two groups: one for possible changes that occurred in burned areas over the ten-year period and the other for remaining burned polygons where the initial screening suggested no change.

Tables S.4 and S.5 report the Landsat-5 and -8 spectral bands, their width, and spatial resolutions, respectively. In particular, the NIR band corresponds to Band-4 for Landsat-5 and Band-5 for Landsat-8, while the SWIR band is associated with the *SWIR-1 band* for both Landsat-5 (Band-5) and Landsat-8 (Band-6).

This initial screening procedure compares the yearly variation of two optical indexes averaged within the polygon of each burned area with the mean values obtained in its surroundings. The procedure is based on the computation of the Normalised Difference Built-up Index (NDBI) and the Surface Reflectance-derived Normalized Difference Vegetation Index (NDVI). The possible changes alert is not activated when a burned polygon does not exceed a difference in the empirically defined threshold for NDVI or NDBI (annual absolute difference < 0.25). In this case, since no change occurred during the period considered, the polygon was filtered out from the dataset. The output of the described approach produced a binary mask result for each polygon and, by comparing each year to the next, assigned either a 0 or a 1 to each polygon, where the value 1 signals a burned area where a possible change took place and the value 0 otherwise, i.e., indicating that the threshold was not exceeded.

In the screening phase, all polygons smaller than 10 hectares, associated with no changes by the algorithm, were filtered out of the dataset. The remaining burned areas of a size smaller than 10 hectares with a suspected change, along with all burned areas 10 hectares or larger, were then analyzed using the visual inspection-based approach described in section **B.3** to ensure the robustness of the procedure that generates our violation dataset.

The initial filtering step was crucial to make the work feasible within an acceptable time frame, as the visual inspection phase is extremely time-consuming and we started out with thousands of burned polygons to monitor, the majority being smaller than 10 hectares.

Table S.3. Satellite products used to perform the analysis

Landsat-5			
Year	Satellite/sensor	Imagery date	Path/Row
2005	Landsat-5/TM	20050719	193/31
2005	Landsat-5/TM	20050719	193/32
2005	Landsat-5/TM	20050719	193/33
2005	Landsat-5/TM	20050813	192/32
2005	Landsat-5/TM	20050813	192/33
2006	Landsat-5/TM	20060823	193/31
2006	Landsat-5/TM	20060823	193/32
2006	Landsat-5/TM	20060823	193/33
2006	Landsat-5/TM	20060731	192/32
2006	Landsat-5/TM	20060731	192/33
2007	Landsat-5/TM	20070709	193/31
2007	Landsat-5/TM	20070709	193/32
2007	Landsat-5/TM	20070709	193/33
2007	Landsat-5/TM	20070718	192/32
2007	Landsat-5/TM	20070718	192/33
2009	Landsat-5/TM	20090815	193/31
2009	Landsat-5/TM	20090730	193/32
2009	Landsat-5/TM	20090730	193/33
2009	Landsat-5/TM	20090723	192/32
2009	Landsat-5/TM	20090808	192/33
2011	Landsat-5/TM	20110906	193/31
2011	Landsat-5/TM	20110906	193/32
2011	Landsat-5/TM	20110906	193/33
2011	Landsat-5/TM	20110713	192/32
2011	Landsat-5/TM	20110713	192/33

Notes: the Path/Row values uniquely define the Landsat tiles along the orbit.

Landsat-8			
Year	Satellite/sensor	Imagery date	Path/Row
2013	Landsat-8/OLI	20130725	193/31
2013	Landsat-8/OLI	20130725	193/32
2013	Landsat-8/OLI	20130725	193/33
2013	Landsat-8/OLI	20130803	192/32
2013	Landsat-8/OLI	20130803	192/33
2014	Landsat-8/OLI	20140813	193/31
2014	Landsat-8/OLI	20140813	193/32
2014	Landsat-8/OLI	20140813	193/33
2014	Landsat-8/OLI	20140806	192/32
2014	Landsat-8/OLI	20140806	192/33
2015	Landsat-8/OLI	20150731	193/31
2015	Landsat-8/OLI	20150731	193/32
2015	Landsat-8/OLI	20150731	193/33
2015	Landsat-8/OLI	20150708	192/32
2015	Landsat-8/OLI	20150708	192/33
2016	Landsat-8/OLI	20160717	193/31
2016	Landsat-8/OLI	20160717	193/32
2016	Landsat-8/OLI	20160820	193/33
2016	Landsat-8/OLI	20160726	192/32
2016	Landsat-8/OLI	20160811	192/33
2017	Landsat-8/OLI	20170805	193/31
2017	Landsat-8/OLI	20170805	193/32
2017	Landsat-8/OLI	20170805	193/33
2017	Landsat-8/OLI	20170729	192/32
2017	Landsat-8/OLI	20170729	192/33
2018	Landsat-8/OLI	20180707	193/31
2018	Landsat-8/OLI	20180707	193/32
2018	Landsat-8/OLI	20180707	193/33
2018	Landsat-8/OLI	20180801	192/32
2018	Landsat-8/OLI	20180801	192/33
2019	Landsat-8/OLI	20190726	193/31
2019	Landsat-8/OLI	20190726	193/32
2019	Landsat-8/OLI	20190726	193/33
2019	Landsat-8/OLI	20190804	192/32
2019	Landsat-8/OLI	20190804	192/33

Table S.4. Landsat 5 Thematic Mapper (TM)

	Spectral Resolution (μm)	Spectrum Range	Spatial Resolution
Band 1	0.441-0.514	VIS (BLUE)	30 m
Band 2	0.519-0.601	VIS (GREEN)	30 m
Band 3	0.631-0.692	VIS (RED)	30 m
Band 4	0.772-0.898	NIR	30 m
Band 5	1.547-1.749	SWIR-1	30 m
Band 6	10.31-12.36	TIR	120 m
Band 7	2.064-2.345	SWIR-2	30 m

Notes: VIS stands for Visible; NIR stands for Near Infrared; SWIR stands for Short-wave infrared; TIR stands for Thermal infrared.

Table S.5. Landsat 8 Operational Land Imager and Thermal Infrared sensor (OLI&TIRS)

	Spectral Resolution (μm)	Spectrum Range	Spatial Resolution
Band 1	0.435-0.451	Aerosol	30 m
Band 2	0.452-0.512	VIS (BLUE)	30 m
Band 3	0.533-0.590	VIS (GREEN)	30 m
Band 4	0.636-0.673	VIS (RED)	30 m
Band 5	0.851-0.879	NIR	30 m
Band 6	1.566-1.651	SWIR-1	30 m
Band 7	2.107-2.294	SWIR-2	30 m
Band 8	0.503-0.676	PAN	15 m
Band 9	1.363-1.384	Cirrus	30 m
Band 10	10.60-11.19	TIR-1	100 m
Band 11	11.50-12.51	TIR-2	100 m

Notes: VIS stands for Visible; NIR stands for Near Infrared; SWIR stands for Short-wave infrared; TIR stands for Thermal infrared; PAN stands for Panchromatic.

We verified the validity of the screening procedure that allowed us to filter out from the final dataset the small burned polygons with no changes. In each year, from a random selection of about 10% of all burned polygons smaller than 10 hectares that were removed from the dataset, we verified the result of the first-step screening procedure by visual inspection. We observed that for these polygons, the algorithm effectively detected the absence of changes with an overall accuracy of 98.9%. Details about this validation activity are reported in Table S.6. Instead, we have chosen to perform the visual inspection described in section **B.3** on all burned polygons greater than 10 hectares, since the change alert implemented in the previous filtering step should not be considered as reliable in this case. This is mainly due to the limited impact of the occurred changes on the mean value of NDVI or NDBI, which is calculated as an average over the entire polygon. In fact, for large polygons, the number of pixels affected by the change produced by an alleged violation is usually negligible compared to the total number of pixels.

Table S.6. Validation process of the algorithm used to filter out polygons of burned areas smaller than 10 hectares associated with no change.

Year	Accuracy	Total polygons	Visually inspected polygons
2005	98.60%	743	74
2006	97.50%	1234	122
2007	97.10%	2406	240
2008	100.00%	996	100
2009	99.20%	2614	260
2010	98.60%	1462	146
2011	99.40%	1622	162
2012	99.00%	1009	100
2013	100.00%	738	73
2014	98.40%	1225	122
2015	100.00%	894	89

B.3 Visual inspection phase

In the final step, we visually inspected all burned polygons greater than 10 hectares and the small burned polygons (less than 10 hectares) flagged by the initial screening. This allows us to determine whether any buildings or infrastructure have been constructed in these areas, indicating a violation. For the visual inspection phase on the final dataset, we used Very High Resolution satellite and aerial images available from the Google Earth Pro Historical Imagery catalog. By leveraging upon the detailed imagery provided by Google Earth Pro, we ensure accurate and reliable results in our analysis.
